# Cellular localization of Arabidopsis EARLY FLOWERING3 is responsive to light quality

**DOI:** 10.1093/plphys/kiac072

**Published:** 2022-02-22

**Authors:** James Ronald, Chen Su, Lei Wang, Seth J Davis

**Affiliations:** Department of Biology, University of York, Heslington, York YO10 5DD, UK; Key Laboratory of Plant Molecular Physiology, CAS Center for Excellence in Molecular Plant Sciences, Institute of Botany, Chinese Academy of Sciences, Beijing 100093, China; University of Chinese Academy of Sciences, Beijing 100049, China; Key Laboratory of Plant Molecular Physiology, CAS Center for Excellence in Molecular Plant Sciences, Institute of Botany, Chinese Academy of Sciences, Beijing 100093, China; University of Chinese Academy of Sciences, Beijing 100049, China; Department of Biology, University of York, Heslington, York YO10 5DD, UK; State Key Laboratory of Crop Stress Biology, School of Life Sciences, Henan University, Kaifeng 475004, China

## Abstract

Circadian clocks facilitate the coordination of physiological and developmental processes to changing daily and seasonal cycles. A hub for environmental signaling pathways in the Arabidopsis (*Arabidopsis thaliana*) circadian clock is the evening complex (EC), a protein complex composed of EARLY FLOWERING3 (ELF3), ELF4, and LUX ARRYTHMO (LUX). Formation of the EC depends on ELF3, a scaffold protein that recruits the other components of the EC and chromatin remodeling enzymes to repress gene expression. Regulating the cellular distribution of ELF3 is thus an important mechanism in controlling its activity. Here, we determined that the cellular and sub-nuclear localization of ELF3 is responsive to red (RL) and blue light and that these two wavelengths have apparently competitive effects on where in the cell ELF3 localizes. We further characterized the RL response, revealing that at least two RL pathways influence the cellular localization of ELF3. One of these depends on the RL photoreceptor phytochrome B (phyB), while the second is at least partially independent of phyB activity. Finally, we investigated how changes in the cellular localization of ELF3 are associated with repression of EC target-gene expression. Our analyses revealed a complex effect whereby ELF3 is required for controlling RL sensitivity of morning-phased genes, but not evening-phased genes. Together, our findings establish a previously unknown mechanism through which light signaling influences ELF3 activity.

## Teaser

Light signaling pathways converge on the evening complex scaffold protein EARLY FLOWERING3 by controlling its cellular and sub-cellular localization and regulating circadian clock gene expression.

## Introduction

Circadian clocks are internal biological timekeeping mechanisms that integrate light, temperature, and other stimuli with predictable daily changes so that internal physiological responses are coordinated with the external day–night cycle. In plants, the circadian clock is responsible for regulating growth, the floral transition, metabolism, and the response to both biotic and abiotic stressors (Inoue et al., 2018). Accordingly, plants whose internal circadian cycle closely follows the external cycle have enhanced fitness and productivity (Dodd et al., 2005).

The plant circadian oscillator is composed of a series of interlocking transcriptional–translational feedback loops (Ronald and Davis, 2017). Within these loops, the evening complex (EC) has been established as a core component of the oscillator required to sustain circadian rhythms, facilitate entrainment to photo and thermal cycles, and control the time of day sensitivity of the oscillator to light and temperature stimuli (Covington et al., 2001; Mcwatters et al., 2007; Thines and Harmon, 2010; Kolmos et al., 2011; Herrero et al., 2012; Herrero and Davis, 2012; Undurraga et al., 2012; Anwer et al., 2020; Zhu et al., 2022). The EC is a tripartite protein complex composed of EARLY FLOWERING3 (ELF3), ELF4, and LUX ARRYTHMO (LUX) (Nusinow et al., 2011; Herrero et al., 2012). ELF3 is proposed as a scaffold protein that recruits ELF4, LUX, and other transcriptional regulators and chromatin-remodeling enzymes to repress gene expression (Huang et al., 2016; Lee et al., 2019; Park et al., 2019; Tong et al., 2020). The EC directly binds to DNA through the activity of LUX, a MYB-domain transcription factor whose DNA binding activity is temperature sensitive (Chow et al., 2012; Box et al., 2015; Silva et al., 2020). Together, the EC regulates the expression of genes associated with the circadian clock, flowering time, hormone signaling, thermomorphogenesis, and abiotic and biotic stress (Ezer et al., 2017).

The localization of ELF3 to the nucleus is critical for its functional activity (Yu et al., 2008; Kolmos et al., 2011; Herrero et al., 2012; Anwer et al., 2014). Arabidopsis (*Arabidopsis thaliana*) ELF3 is proposed to intrinsically localize to the nucleus through a nuclear localization signal (NLS) within the C-terminus of the ELF3 protein (Liu et al., 2001). However, this NLS motif is not conserved in other ELF3-like protein sequences and other NLS motifs could not be identified (Saito et al., 2012). Instead, ELF3 appears to be shuttled to the nucleus by interacting with other proteins. Expressing the middle region of Arabidopsis ELF3 (ELF3-M), which lacked a functional NLS, still resulted in the localization of this construct to the nucleus (Herrero et al., 2012). Furthermore, co-expressing ELF3-M with ELF4 increased the nuclear localization of ELF3-M, while point mutations within this region subsequently reduced the nuclear localization of ELF3 (Kolmos et al., 2011; Anwer et al., 2014). Therefore, ELF3 may be shuttled to the nucleus through proteins that interact with ELF3, such as ELF4.

Within the nucleus, ELF3 is localized either diffusely in the nucleoplasm or concentrated in sub-nuclear domains. Multiple research groups have observed ELF3 localizing to sub-nuclear domains, and these have been assigned different names. First, ELF3 was shown to localize with CONSTITUTIVE PHOTOMORPHOGENIC1 (COP1) and GIGANTEA (GI) in sub-nuclear structures (Yu et al., 2008). The localization of ELF3, GI, and COP1 together was proposed to lead to the proteolytic degradation of ELF3 and GI. ELF3 was then shown to co-localize with ELF4 in sub-nuclear structures called foci at ambient temperatures (Herrero et al., 2012). The co-localization of ELF3 and ELF4 together led to the proposal that foci may be sites of EC activity. In accordance with this, a reduction in the localization of ELF3 to foci correlated with increased expression of EC circadian-target loci (Kolmos et al., 2011; Anwer et al., 2014).

Alongside associating to sub-nuclear structures with evening-phased proteins, ELF3 also localizes in sub-nuclear structures with TANDEM ZINC-FINGER PLUS3 (TZP), a morning-phased protein (Loudet et al., 2008; Kaiserli et al., 2015). The localization of TZP to nuclear bodies was dependent on the red-light (RL) sensor phytochrome B (phyB), suggesting that ELF3, TZP, and phyB may co-localize together in sub-nuclear structures in the early morning (Kaiserli et al., 2015). Finally, a prion-like domain within the C-terminus of ELF3 was recently demonstrated to mediate phase separation of ELF3 into nuclear speckles in response to warm temperature (Jung et al., 2020), although the effect of temperature on ELF3 sub-nuclear localization is seemingly time dependent (Murcia et al., 2021; Ronald et al., 2021). Therefore, ELF3 appears capable of localizing to multiple different sub-nuclear structures depending on protein–protein interactions, and temporal and environmental factors.

Alongside being a putative thermosensor, light-signaling pathways also converge on ELF3. The N-terminus of ELF3 facilitates a physical interaction with the phyB (Liu et al., 2001). The outcome of the phyB-ELF3 interaction is complex; previous studies have highlighted that the binding of phyB to ELF3 may repress the circadian function of the EC (Kolmos et al., 2011; Herrero et al., 2012), while other studies have revealed that phyB stabilizes ELF3 (Nieto et al., 2015) and connects ELF3 to a wider network of light-signaling associated proteins (Huang et al., 2016). Alongside phyB, other proteins that interact with ELF3 may indirectly connect ELF3 to other light signaling pathways. For example, ELF4 binds to the middle region of ELF3 and regulates the nuclear and sub-nuclear localization of ELF3 (Kolmos et al., 2011; Herrero et al., 2012; Anwer et al., 2014). The expression of *ELF4* is directly regulated by RL, UV-B, and far-red (FRL) light-signaling pathways (Tepperman et al., 2001; Fehér et al., 2011; Siddiqui et al., 2016). In this way, light induction of ELF4 is hypothesized to facilitate activation of ELF3.

Here, we further investigated how light signaling governs ELF3 activity. We observed that the cellular localization of ELF3 is responsive to pulses of RL and blue light (BL), and these wavelengths have opposite, and potentially competing effects on the sub-nuclear distribution of ELF3. We also identified phyB-dependent and phyB-independent effects of RL on ELF3, indicating other RL photoreceptors must also regulate ELF3 localization. Finally, we investigated the effect of these light pulses and the *phyB* mutation on the ability of ELF3 to regulate gene expression. This analysis identified a requirement for ELF3 in regulating the sensitivity of the morning-phased *PSEUDO RESPONSE REGULATOR9* (*PRR9*) to RL pulses. Together, this work reveals a mechanism for how light signaling influences the activity of ELF3.

## Results

### The localization of ELF3 is responsive to light

**Figure 1:**
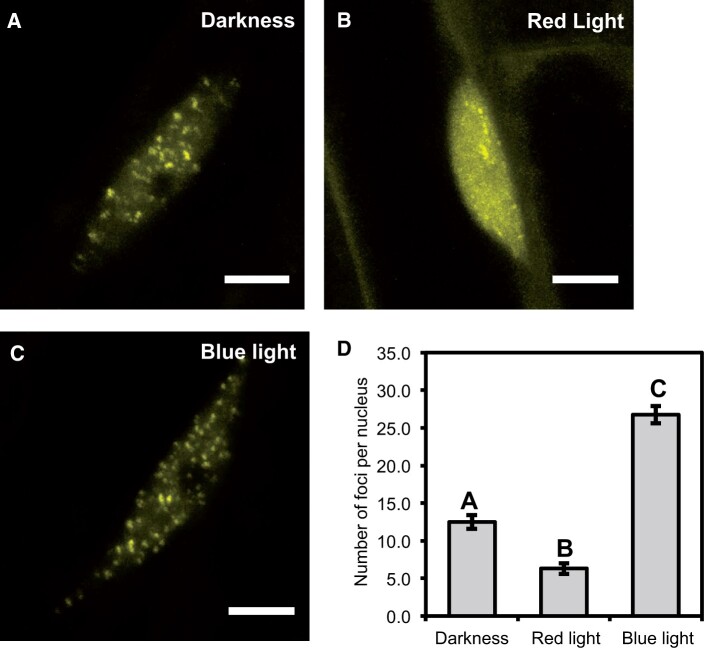
The sub-nuclear localization of ELF3 is responsive to light. The localization of *35S::YFP:ELF3* in hypocotyl nuclei after samples were either transferred to the (A) dark or after a 25 µmol (B) RL or (C) BL pulse. All light pulses were started at ZT7 (short-day 6/18 photoperiods) and applied for 3 h before imaging was started at ZT10. As a control, samples were transferred to the dark at ZT7. Scale bars equal 5 µm. D, Mean number of foci per nucleus under the respective light treatments. Error bars are standard error of the mean. A minimum of 11 nuclei were analyzed in total for each light treatment with images collected from multiple biological samples. A one-way ANOVA with a Tukey-HSD post hoc test was used to determine significance, different letters signify a significant difference of *P* < 0.001.

We first tested whether the cellular distribution of ELF3 to foci was responsive to light. Arabidopsis seedlings expressing *35S::YFP:ELF3* (previously described in Herrero et al., 2012 and henceforth referred to as ELF3) were pulsed for 3 h with 25 µmol/m^−2^/s^−1^ (µmol henceforth) of RL or BL at ZT7 (*zeitgeber* time) and then immediately imaged at ZT10. As a control, ELF3 seedlings were transferred to the dark for the duration of the light pulse. Regardless of the light treatment, there was at least one focus per hypocotyl nuclei in all nuclei that were imaged (Figure 1). Direct measurements of foci morphology were attempted, but it was not possible to accurately resolve the boundary between the nucleoplasm and focus. Hence, further quantification was not attempted and instead we just counted the number of foci per nucleus. In the dark, there were ∼12 foci per nucleus (Figure 1, A and D). After a RL pulse, the number of foci was reduced to ∼6 foci per nucleus, while after a BL pulse the number of foci increased to ∼25 per nucleus (Figure 1C). There was no noticeable change in the amount of nucleoplasmic signal between samples transferred to the dark or those pulsed with BL, while in samples pulsed with RL there was a clear increase in the nucleoplasmic signal compared to nuclei either transferred to the dark or pulsed with BL (Figure 1, A–C). Comparing the morphology of foci under the respective treatments did not reveal any consistent effect of BL pulse on ELF3 foci morphology, while foci that were pulsed with RL appeared smaller than those in the dark or pulsed with BL (Figure 1, A–C). Together, this revealed that the sub-nuclear dynamics of ELF3 are light responsive.

The observed effects of RL and BL on ELF3 foci dynamics could be indirect and instead reflect changes in the nuclear/cytoplasmic (N/C) partitioning and/or stability of ELF3. To test this, we first measured the N/C ratio of ELF3 in the dark or after the respective light pulses described above. Under all light treatments, ELF3 was localized to the nucleus and cytoplasm (Supplemental Figure S1, A–C). The cellular distribution was modestly influenced by a light pulse. There was a small (∼9%), although not statistically significant, decrease in the N/C ratio of ELF3 following a RL pulse compared with samples in the dark (Supplemental Figure S1D). In contrast, a BL pulse caused a sizeable increase (∼50%) in the N/C ratio of ELF3 (Supplemental Figure S1D). Comparing the relative nuclear and total signal revealed that a RL pulse reduced the nuclear signal but caused no change to the normalized total signal, while for BL both the nuclear and total signal was increased compared to samples in the dark (Supplemental Figure S1, E and F).

To confirm that the effects of RL on the sub-nuclear distribution of ELF3 occurred independently of changes in the stability of ELF3, we analyzed total protein levels of ELF3 under the different light treatments. There was no discernible difference in the total protein levels of ELF3 between samples transferred to the dark or pulsed with RL (Supplemental Figure S2). Furthermore, there was also no discernible changes in the stability of ELF3 following a BL pulse compared to the darkness control (Supplemental Figure S2). In summary, the cellular localization of ELF3 is responsive to light: RL pulses suppress the sub-nuclear localization of ELF3, while BL pulses result in the nuclear and sub-nuclear localization of ELF3 increasing.

### Red and BL have a competitive effect on ELF3 localization

As RL and BL caused opposite effects on the localization of ELF3, we next investigated whether there was competition between RL and BL on ELF3 localization. As the effect of both RL and BL was more strongly observed on the sub-nuclear localization of ELF3 foci, we characterized the response of ELF3 foci to light pulses with different spectral ratios of RL and BL. Seedlings were pulsed with either equal ratios of RL and BL (25 µmol:25 µmol), predominantly BL (40 µmol BL:20 µmol RL) or predominantly RL (25 µmol RL:12 µmol BL). Together, these white light (WL) pulses were termed WL(=), WL(BL+), and WL(RL+), respectively. The application of the light pulses was carried out as described above.

**Figure 2:**
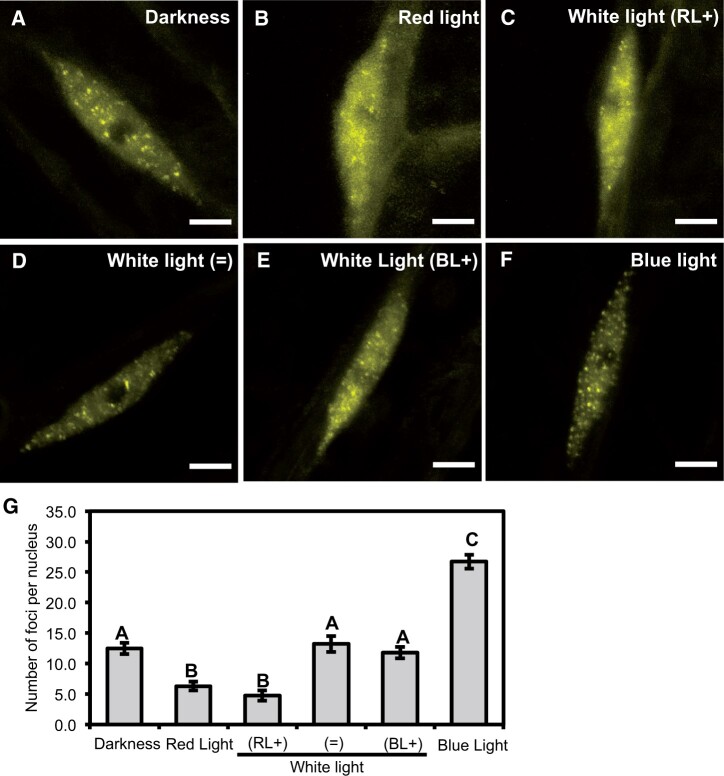
Light has a competitive effect on the sub-nuclear localization of ELF3. Hypocotyl nuclei of seedlings expressing *35S::YFP:ELF3* under different light treatments at ZT10 (short-day 8/16 photoperiods). A, Darkness; B, 25 µmol monochromatic RL; C, WL: 25 µmol of RL and 12 µmol of BL; D, WL: 25 µmol of RL and 25 µmol of BL; E, WL: 20 µmol of RL and 40 µmol of BL; and F, 25 µmol monochromatic BL. Scale bars are 5 µm. All light pulses were applied for 3 h starting at ZT7. G, Mean number of foci per nucleus under the respective light treatments. Error bars are standard error of the mean. A minimum of 11 nuclei were imaged for each light treatment with images collected from multiple biological samples. Significance was determined by a one-way ANOVA with a Tukey HSD post hoc test. Different letters signify significant difference of *P* < 0.001. The darkness, monochromatic BL, and RL datasets are the same as presented in Figure 1.

ELF3 was localized between the nucleoplasm and foci regardless of the spectral composition of the WL pulse (Figure 2). However, the RL:BL ratio did influence the number of foci that were observed. In samples pulsed with WL(=) the number of foci was similar number to the foci observed in nuclei of seedlings transferred to the dark (Figure 2, A, D, and G). However, there was a large amount of biological variation in the number of foci after a WL(=) pulse. The source of this variation was unclear; foci formation after the WL(=) pulse was highly variable both within and across different biological samples that were measured on separate occasions. The number of foci observed following a WL(=) pulse reflected the spectrum of responses seen in nuclei pulsed with monochromatic RL or BL, or in those transferred to the dark (Supplemental Figure S3, A–G). After a WL(RL+) pulse, the number of foci per nuclei was reduced and the nucleoplasmic signal increased (Figure 2, C and G). The effect of a WL(RL+) pulse was similar to the effect we observed previously for a monochromatic RL pulse.

To confirm that the BL intensity used in the WL(RL+) could promote foci formation, we analyzed the number of foci per nuclei after a 12-µmol monochromatic BL pulse. As with a 25-µmol monochromatic BL pulse, a 12-µmol monochromatic BL pulse promoted foci formation and there was no significant difference in the effect of a 12-µmol BL pulse compared with a 25-µmol BL pulse (Supplemental Figure S4, A–C and E). This suggests that RL can directly suppress the effect of BL on promoting ELF3’s localization to foci. Supporting this, the number of foci after a WL(BL+) was slightly, but not statistically significantly, reduced compared with samples transferred to the dark. As with a 12- or 25-µmol pulse of BL, a 40-µmol monochromatic BL pulse (the intensity of BL used in the WL(BL+) pulse) was sufficient to promote foci formation (Supplemental Figure S4, D–E). Thus, RL suppress the effect of BL on the sub-nuclear localization of ELF3.

### RL has a dosage-dependent effect on ELF3 sub-nuclear localization

The *phyB* circadian phenotype is dependent on the intensity of RL. Below intensities of 10 µmol RL, the *phyB* mutant has no discerned circadian phenotype, while increases in the intensity >10 µmol progressively increased the severity of the *phyB* phenotype (Somers et al., 1998). To determine whether sub-nuclear localization of ELF3 was responsive to different intensities of RL, we investigated the localization of ELF3 after a 3-h long 1, 10, or 15 µmol of RL pulse. The timing and duration of the light pulse was carried out as described above. This dataset is compared to the darkness and RL25 dataset (Figure 1).

**Figure 3:**
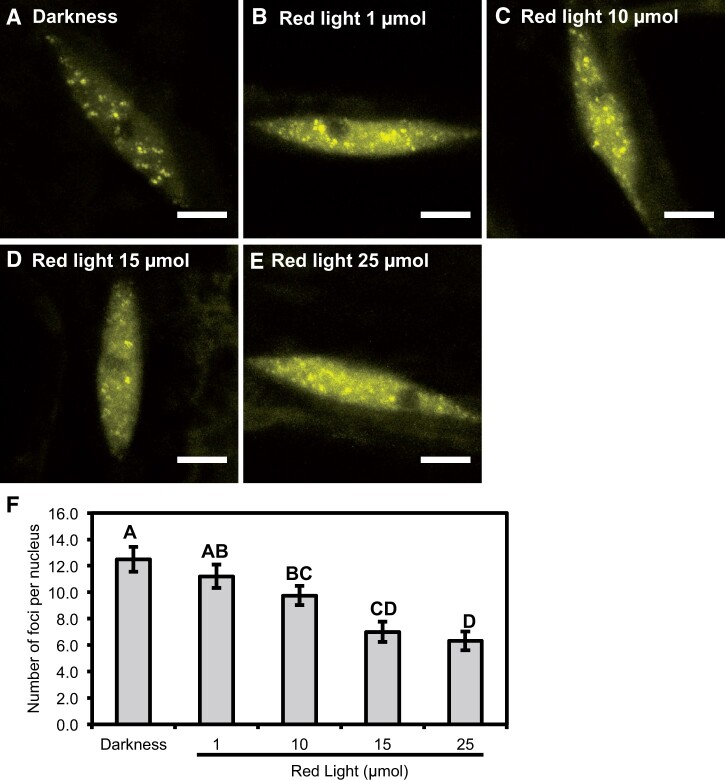
The sub-nuclear localization of ELF3 is responsive to RL in a dosage-dependent manner. The nuclear localization of *35S::YFP:ELF3* (*elf3-4*) in the (A) dark, or after a (B) 1 µmol, (C) 10 µmol, (D) 15 µmol, or (E) 25 µmol RL pulse. Scale bars are equal to 5 µm. F, Mean number of foci per nucleus for *35S::YFP:ELF3* (*elf3-4*) under the respective light treatment. Error bars are standard error of the mean. All light pulses were started at ZT7 (short-days) and carried out for 3 h before seedlings were imaged at ZT10. A minimum of 12 images were analyzed for each light treatment with images collected from multiple biological samples. Significance was determined by one-way ANOVA with a Tukey HSD post hoc test. Different letters signify a significance of *P* < 0.05. The data for darkness and 25 µmol RL data ARE the same as Figure 1.

Across all tested intensities of RL, ELF3 was localized between the cytoplasm and nucleus, and localized to nuclear foci (Figure 3). However, the number of foci per nucleus was dependent on the intensity of RL. A 1-µmol RL pulse caused a slight, but not significant, decrease in the number of foci per nucleus compared with nuclei in the dark (Figure 3, A, B, and F). Further increases in the intensity of the RL pulse resulted in a stronger inhibitory effect. A 10-µmol RL pulse reduced the number of foci per nucleus to ∼10, while a 15-µmol RL pulse resulted in a further reduction in the number of foci to ∼7 (Figure 3, C and D). The effect of a 15-µmol RL pulse was comparable to the effect caused by a 25-µmol RL pulse (∼6 foci on average) (Figure 3E). Unlike the 25-µmol RL pulse, there was no effect of the 10 or 15 µmol RL pulse on the N/C ratio, relative nuclear or relative total signal of ELF3 (Supplemental Figure S5, A–F). Together, these results suggest that RL has a dosage-dependent effect on the association of ELF3 to foci that occurs independently of changing the N/C distribution of ELF3.

### phyB promotes the nuclear and sub-nuclear localization of ELF3

To identify whether phyB directly regulated the cellular localization of ELF3, the *35S::YFP:ELF3 elf3-4* transgenic line was introgressed into the *elf3-4/phyB-10* double mutant to generate a stable *35S::YFP:ELF3 elf3-4/phyB-10* line. This line will henceforth be referred to as ELF3 (B−), while the line with a wild-type (WT) *phyB* allele will be referred to as ELF3 (B+). The cellular and sub-nuclear localization of ELF3 (B−) was then imaged in seedlings either transferred to the dark or pulsed with 25 µmol RL following the approach described above.

**Figure 4:**
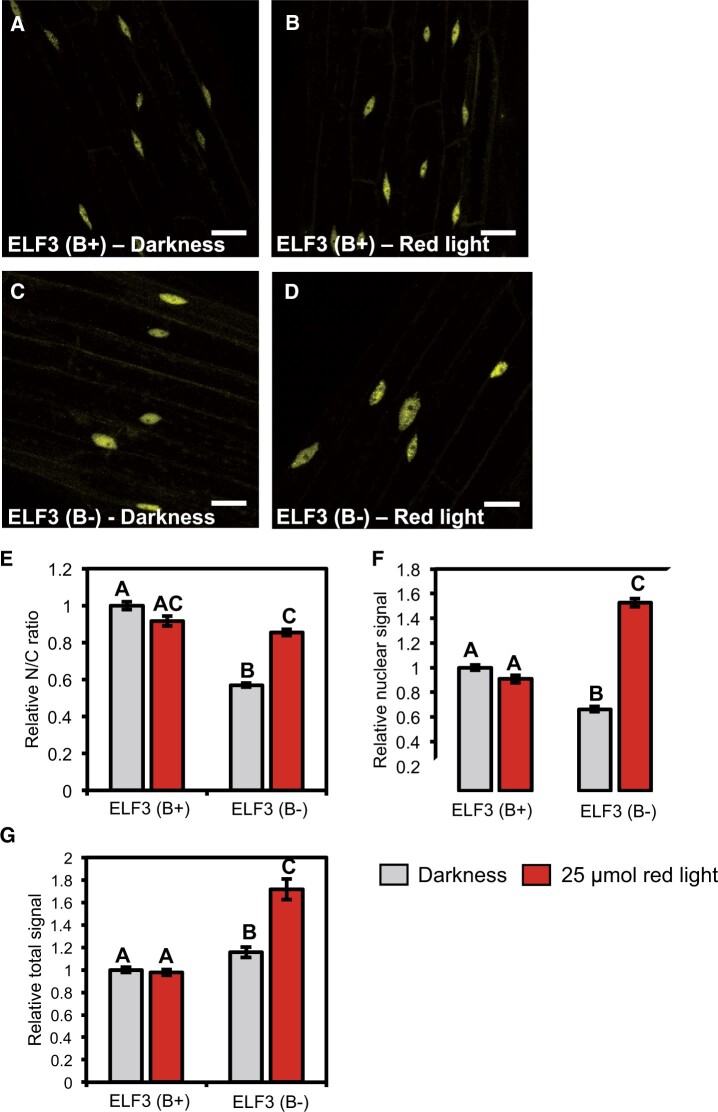
Phytochrome B promotes the nuclear localization of ELF3. The localization of *35S::YFP:ELF3* in hypocotyl cells of either the (A–B) *elf3-4* and (C–D) *elf3-4/phyB-10* mutant. Images are from the (A, C) dark or (B, D) after a 25-µmol RL pulse. Light pulses were started at ZT7 before images were collected at ZT10. Scale bars represent 25 µm. E, Relative N/C ratio; F, relative nuclear signal; and G, relative total signal of the respective constructs in the dark or after a 25-µmol RL pulse. All data were made relative to the values of *35S::YFP:ELF3* (*elf3-4*) in the dark at ZT10. Error bars are standard error of the mean. A minimum of six images were analyzed for each light treatment. The values for *35S::YFP:ELF3* (*elf3-4*) are the same as first presented in Supplemental Figure S1. Different letters signify significance difference (*P* < 0.01) as determined by a one-way ANOVA with a Tukey HSD post hoc test.

As with ELF3 (B+), ELF3 (B−) was localized to the cytoplasm and nucleus in the dark. However, the nuclear accumulation of ELF3 was severely compromised by the *phyB* mutation. The N/C ratio of ELF3 (B−) in the dark was decreased by ∼40% compared with ELF3 (B+) (Figure 4, A, C, E, and F). The localization of ELF3 (B−) remained responsive to RL but displayed the opposite response to ELF3 (B+). Instead of decreasing, the nuclear localization of ELF3 (B−) increased after a RL pulse (Figure 4, B and D). The N/C ratio of ELF3 (B−) following a RL pulse was comparable to the N/C ratio of ELF3 (B+) after a RL pulse. However, the relative nuclear signal of ELF3 (B−) after a RL pulse was greater than the nuclear signal of ELF3 (B+) samples in the dark (Figure 4F). The disparity in the N/C ratio and nuclear signal of ELF3 (B−) was caused by changes in the total signal of ELF3 (B−). In the dark and after a RL pulse, the relative total signal of ELF3 (B−) was greater than ELF3 (B+) (Figure 4G). ELF3 (B−) total signal in the dark only displayed a small increase (∼15%), while a RL pulse caused a large increase (∼71%) relative to the total signal of ELF3 (B+) in the dark, respectively.

**Figure 5:**
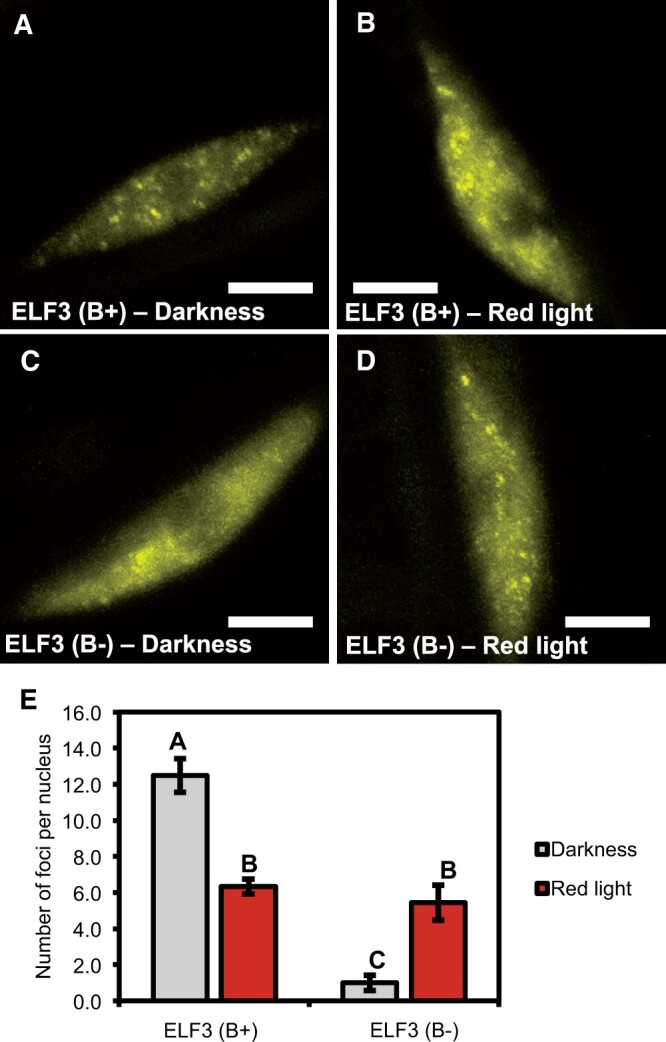
phyB is required for ELF3 to localize to foci. The localization of *35S::YFP:ELF3* in hypocotyl nuclei of either the (A–B) *elf3-4* or (C–D) *elf3-4/phyB-10* mutant. Samples were either transferred to the (A, C) dark or (B, D) pulsed with 25 µmol RL pulse for 3 h at ZT7 before images were collected at ZT10. Scale bars represent 5 µm. E, Mean number of foci per nucleus for the respective construct and light treatment. A minimum of 12 images were analyzed for each light treatment. Error bars are standard error of the mean. Significance was determined by a one-way ANOVA with a Tukey HSD post hoc test to determine significance. Different letters signify a significant difference of *P* < 0.001. The values for *35S::YFP:ELF3* (*elf3-4*) are the same as first presented in Figure 1.

The *phyB* mutation also had a strong phenotypic effect on the localization of ELF3 to foci (Figure 5, A–D). In the dark, ELF3 (B−) was predominantly found to be localized to the nucleoplasm (Figure 5C). When foci were observed, the number of foci per nucleus was strongly reduced; Excluding the 50% of nuclei (*n* = 12) that had no focus, there was ∼2 foci per nucleus for ELF3 (B−) in the dark (Figure 5E). In comparison, ELF3 (B+) had ∼12 and ∼6 foci per nucleus in the dark or after a RL pulse, respectively. Alongside reducing the number of foci, ELF3 (B−) foci were also smaller and less intense than ELF3 (B+) both in the dark and after a RL pulse (Figure 5, A–C). As with the cellular localization of ELF3, the sub-nuclear localization of ELF3 (B−) remained responsive to RL, but again, displayed the opposite response to ELF3 (B+) (Figure 5, B, D, and E). Both the incidence of nuclei with at least one focus and the number of foci observed per nucleus increased after a RL pulse: 89% of all ELF3 (B−) nuclei had at least one focus following a RL pulse (*n* = 18), while the mean number of foci per nucleus increased to ∼5. However, the number of foci per nucleus in ELF3 (B−) after a RL pulse was highly variable, with the number of foci varying from 1 to 12 per nucleus (Figure 5E). ELF3 (B−) foci after a RL pulse were also larger and brighter than those observed in the dark and more closely resembled the foci of ELF3 (B+). Therefore, we found that phyB has an essential role in facilitating the cellular localization of ELF3, but other phytochromes, such as phyA, may also contribute in regulating the localization of ELF3.

### Photoactivated phys are required for the nuclear accumulation of ELF3

To confirm that phys were directly regulating the cellular and sub-nuclear localization of ELF3, ELF3 (B+) seedlings were pulsed with FRL. A FRL pulse promotes the rapid photoconversion of phys from their active Pfr form to the inactive Pr form (Van Buskirk et al., 2014). Seedlings were either pulsed with 110 µmol of FRL for 15 min or were kept under WL for the duration of the FRL pulse. Under WL and FRL, ELF3 (B+) was localized between the cytoplasm and nucleus but, the N/C ratio and relative nuclear signal of ELF3 was reduced in seedlings pulsed with FRL (Supplemental Figure S6, A–D). There was no change in the normalized total signal between the two light treatments (Supplemental Figure S6E), supporting that the changes in the N/C ratio and nuclear signal are reflective of changes in the cellular partitioning of ELF3. Within the nucleus, ELF3 was distributed to the nucleoplasm and foci at ZT7 under both light treatments (Supplemental Figure S6, F and G). However, the number of foci was reduced following a FRL pulse (Supplemental Figure S6H). There was no clear morphological change in the appearance of foci pulsed with FRL or kept under WL. Furthermore, for both light treatments foci at ZT7 were smaller and less bright than foci observed at ZT10, similar to our previous results (Ronald et al., 2021). In summary, these results support our hypothesis that photoactivated phys are required for the nuclear accumulation of ELF3.

### ELF3 regulates RL induction of *PRR9* expression

To understand whether the cellular or sub-nuclear changes in the localization of ELF3 following the RL pulse and *phyB* mutation were associated with changes in the functional activity of ELF3, we measured the expression of genes that have been established as targets of ELF3. The expression of ELF3 targets was measured in a WT, ELF3 (B+), and ELF3 (B−) background at ZT10 either in the dark or after a 25-µmol RL pulse. The RL pulse was applied as described above.

**Figure 6:**
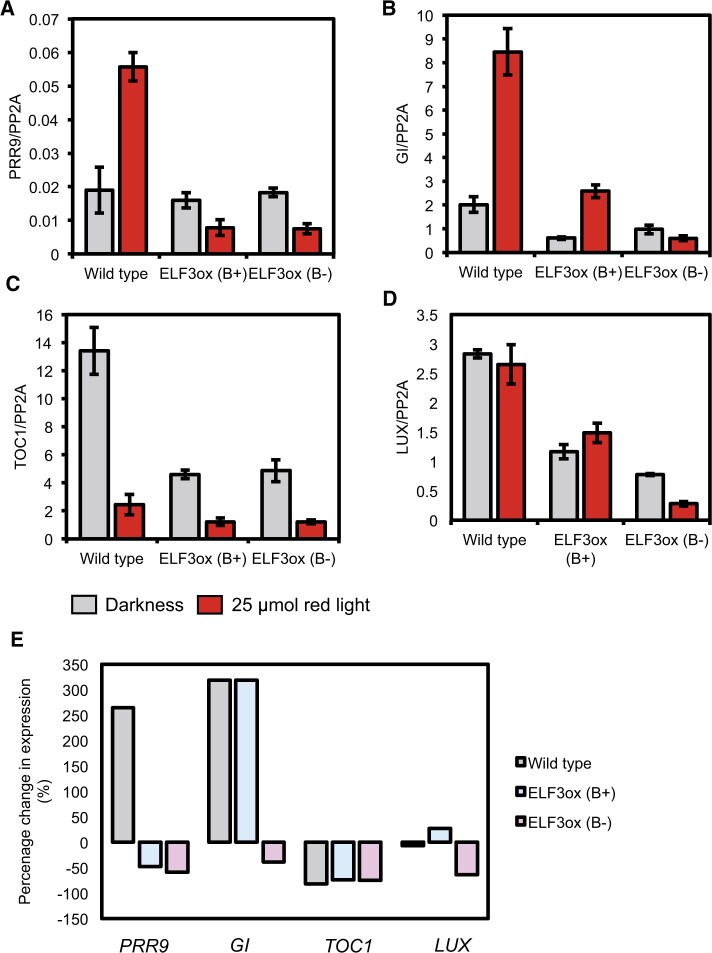
Complex effect of phyB in regulating ELF3 transcriptional activity. The expression of (A) *PRR9* (B) *GI* (C) *TIMING OF CAB1 EXPRESSION* (*TOC1*), and (D) *LUX ARRYTHMO* (*LUX*) was measured in WT (Ws-2), *35S::YFP:ELF3 elf3-4* (ELF3ox B+), or *35S::YFP:ELF3 elf3-4/phyB-10* (ELF3ox B-) at ZT10. Respective lines were either prior pulsed for 3 h with 25 µmol of RL (red bars) or transferred to the dark (gray bars) at ZT7 as a control. Data were normalized to the expression of *PP2A*. The presented data are the mean of at least two technical replicas. Error bars indicate standard deviation among the technical replicas. These results are representative of two biological replicas. E, The relative change in the expression of *PRR9*, *GI*, *TOC1*, and *LUX* following a RL pulse compared to the expression of the respective gene in the dark at ZT10.

We first focused on the expression of *PRR9*, as the expression of *PRR9* is RL-responsive and phyB was proposed to inhibit ELF3’s ability to repress the expression of *PRR9* (Ito et al., 2007; Herrero et al., 2012). At ZT10 in the dark, the expression of *PRR9* in either ELF3 (B+) or ELF3 (B−) was unchanged compared to WT (Figure 6A), consistent with earlier work (Nieto et al., 2015). The expression of *PRR9* was strongly induced by a RL pulse in the WT background (Figure 6E). In contrast, the expression of *PRR9* was not induced by a RL pulse in either the ELF3 (B+) or ELF3 (B−) background. Instead, the expression of *PRR9* decreased in both backgrounds following a RL pulse (Figure 6, A and E). There was no difference in the relative change of *PRR9* expression between ELF3 (B+) and ELF3 (B−), suggesting that ELF3’s ability to antagonize RL-induction of *PRR9* expression is not repressed by phyB in the evening.

ELF3/EC also regulates the expression of other genes that are also light responsive. Amongst these is *GI*, an evening-phased gene whose expression was found to be induced by RL (Molas et al., 2006). The expression of *GI* was repressed in ELF3 (B+) and ELF3 (B−) compared with WT at ZT10 in the dark, although the repressive effect of ELF3 (B−) was weaker than ELF3 (B+) (Figure 6B). As previously reported, a RL pulse strongly promoted the expression of *GI* in WT seedlings. The expression of *GI* was also induced in response to the RL pulse in ELF3 (B+), but not ELF3 (B−) where *GI* expression decreased slightly following a RL pulse (Figure 6B). Comparing the relative change in *GI* expression in response to RL between WT and ELF3 (B+) revealed a similar degree of induction in the two backgrounds (Figure 6E). Therefore, ELF3 does not repress RL-induction of *GI* expression.

We also measured the expression of *LUX* and *TIMING OF CAB1 EXPRESSION* (*TOC1*), further direct targets of ELF3/EC repressive activity (Helfer et al., 2011; Lee et al., 2019). As previously reported, the expression of *TOC1* was repressed in ELF3 (B+) (Figure 6C;Lee et al., 2019). *TOC1* expression was also repressed in ELF3 (B−) and there was no significant difference in the repressive activity when compared to ELF3 (B+). A RL pulse strongly repressed the expression of *TOC1* in WT, ELF3 (B+), and ELF3 (B−), and there was no clear difference in the degree of repressive effect between the three genotypes (Figure 6E). The expression of *LUX* in the dark was also repressed in ELF3 (B+) and ELF3 (B−) compared to WT (Figure 6D). Here, ELF3 (B−) exerted a stronger repressive effect than ELF3 (B+) on repressing *LUX* expression (Figure 6D). The expression of *LUX* was not changed by a RL pulse in WT, while the expression of *LUX* increased and decreased slightly in ELF3 (B+) and ELF3 (B−) after a RL pulse, respectively (Figure 6E). Together, these results highlight a complex role for phyB in regulating the transcriptional activity of ELF3 in the evening.

## Discussion

Sensing and integrating environmental signals enhance an organism’s fitness and productivity by allowing endogenous processes to be coordinated with the external environment. Within this, controlling a protein’s cellular localization has become established as a critical regulatory mechanism for light and temperature signaling pathways in plants (Herrero and Davis, 2012; Ronald and Davis, 2019; Hahm et al., 2020). Recently, temperature dependent re-localization of ELF3 within the nucleus was demonstrated to provide a mechanism through which plants sense warm temperature (Jung et al., 2020; Murcia et al., 2021). Here, we have demonstrated that light also regulates the cellular localization of ELF3. RL, FRL, and BL pulses all regulated the cellular and sub-nuclear localization of ELF3 (Figures 1 and 6;Supplemental Figures S1 and S6). There was also a competitive effect of RL and BL on the sub-nuclear localization of ELF3, with the effect of RL suppressing the effect of BL (Figure 2;Supplemental Figures S3 and S4). Therefore, multiple light signaling pathways converge to competitively regulate where ELF3 is localized in the cell.

ELF3 is a large scaffold protein that has been demonstrated to interact with numerous proteins connected directly or indirectly with light signaling (Liu et al., 2001; Huang et al., 2016). So far, no BL photoreceptors have been shown to directly or indirectly interact with ELF3, including in a non-biased mass-spectrometry screen (Huang et al., 2016). This suggests that BL signals to ELF3 indirectly through other proteins that interact with ELF3. For RL and FRL, phyB physically interacts with the N-terminus of ELF3 and it has been suggested that this provides an interface through which RL regulates ELF3 activity (Liu et al., 2001; Herrero et al., 2012). However, we have observed that RL still influenced the spatial distribution of ELF3 in the *phyB* mutant background (Figures 4 and 5). Furthermore, the effect of the RL pulse in the *phyB* background was opposite to the effect observed in a WT background (Figures 1, 4, and 5;Supplemental Figure S1). Together, we propose that RL signals to ELF3 through two separate pathways: a phyB-dependent pathway that suppresses the nuclear and sub-nuclear localization of ELF3, and a secondary pathway required for the nuclear accumulation of ELF3 at dusk that is at least partially independent of phyB.

These apparently contradictory effects of RL could be explained through phy-dependent regulation of *ELF4*. ELF4 is a small, mobile protein that is proposed to function as an activator of ELF3 and EC activity (Kolmos et al., 2011; Anwer et al., 2014; Chen et al., 2020; Jung et al., 2020; Silva et al., 2020). Though the molecular mechanism through which ELF4 activates ELF3 activity is unclear, co-expressing ELF4 and ELF3 together in *Nicotiana benthamiana* mesophyll cells increased the nuclear localization of ELF3 (Herrero et al., 2012). Furthermore, mutations in the ELF4 binding domain of ELF3 reduced the nuclear and sub-nuclear localization of ELF3 in Arabidopsis (Kolmos et al., 2011; Anwer et al., 2014). The expression of *ELF4* is directly activated by RL and FRL in a phyB-dependent and phyA-dependent manner, respectively (Tepperman et al., 2001; Siddiqui et al., 2016). Furthermore, the ability of RL to promote the expression of *ELF4* is gated to occur only at dusk (Siddiqui et al., 2016). Therefore, RL may have a temporal effect on ELF3. In the early morning, phyB directly suppresses ELF3 activity through binding to the N-terminus of ELF3. Conversely at dusk, phyB, and possibly other phys functioning in a redundant manner, activate *ELF4* expression leading to resultant ELF4 protein increasing the nuclear and sub-nuclear accumulation of ELF3.

The role of foci in facilitating ELF3 functional activity remains unclear. Previously the localization of ELF3 to foci was suggested to be sites of ELF3/EC transcriptional activity (Herrero et al., 2012). However, the data we have presented would suggest that these foci are not sites of transcriptional activity. Though a RL pulse suppressed the localization of ELF3 (B+) to foci, there was no clear consistent effect of a RL pulse on the ability of ELF3 (B+) to repress gene expression. Furthermore, ELF3 (B−) still repressed the expression of *GI*, *LUX*, and *TOC1* to a similar level as ELF3 (B+) in the dark, even though the localization of ELF3 to foci in the *phyB* mutant background was strongly impaired (Figures 5 and 6). Therefore, we have observed no direct connection with the ability of ELF3 to regulate gene expression and the localization of ELF3 to foci.

Instead, foci could represent storage sites of ELF3 protein. ELF3 protein is less stable in the dark than the light. It was reported that this is caused by interactions with COP1 at ambient temperatures and XB3 ORTHOLOG 1 IN ARABIDOPSIS THALIANA (XBAT31) and XBAT35 at warm temperatures (Yu et al., 2008; Nieto et al., 2015; Zhang et al., 2021a; 2021b). As the expression and period of ELF3 activity is phased to occur in the evening (Nusinow et al., 2011), mechanisms must exist to stabilize ELF3 proteins in the dark. The localization of phyB to sub-nuclear structures called photobodies was shown to prolong the activity of phyB by preventing light and thermal reversion of phyB into an inactive isoform (Van Buskirk et al., 2014; Legris et al., 2016). Supporting a storage role for ELF3, the work of Murcia et al. (2021) reported reduced stability of ELF3 protein under conditions in which the localization of ELF3 to sub-nuclear structures was reduced. Although we did not see an immediate change in the stability of ELF3 following a RL treatment (Supplemental Figure S2), it would be of interest to understand how protein dynamics of ELF3 changes across the evening following exposure to different pulses of light.

In summary, we have found that light signaling pathways converge on ELF3 through controlling the cellular and sub-nuclear localization of ELF3. Together, these results provide an interface to understand how light signals could influence the activity of the plant circadian clock and subsequently facilitate the mechanism of entrainment. We also highlight the recent work of Jung et al. (2020). Here, the authors reported that ELF3 localizes to speckles in Arabidopsis root nuclei and yeast cells upon heat stress (Jung et al., 2020). Therefore, it is possible that regulating the sub-nuclear localization of ELF3 is a general mechanism through which environmental signaling pathways control the activity of ELF3.

## Materials and methods

### Plant lines

All Arabidopsis (*A.thaliana*) lines used here are in the Wassilewskija-2 (Ws-2) or Columbia-0 (Col-0) background. The *elf3-4 LHY:LUC*, *35S::YFP:ELF3* (*elf3-4*, Ws-2), *phyB-10* (Ws-2) and *35S::GFP:ELF3* (Col-0) lines have all been described previously (Reed et al., 1993; Hicks et al., 1996; Herrero et al., 2012). The *35S::YFP:ELF3* (*elf3-4/phyB-10*) line was generated by crossing the *35S::YFP:ELF3* (*elf3-4*) line into the *elf3-4/phyB-10* double mutant that was generated during the course of this work. The genotyping primers used in this work are described in the Supplemental Material (Supplemental Table S1).

### Light pulse assays

Seeds of the appropriate line were surface sterilized and plated onto 1× Murashige and Skoog (MS) plates with 1.5% w/v phytoagar, 0.25% w/v sucrose and 0.5 g/L MES with a pH of ∼5.7. The top quarter of the MS agar plate was removed to allow seedlings to grow vertically. Seeds were then stratified at 4°C for 3 days before being transferred to a growth chamber with 85 µmol of WL and a constant temperature of 22°C. Seedlings were then grown vertically for 6 days under a short-day (8/16) photoperiod. Experiments were started at ZT7 (1 h before dusk) on Day 7. Seedlings were transferred to a custom-built LED stack and pulsed with RL, BL, or FRL at the intensities and duration stated in the text. For RL and BL pulses, control plates were transferred to the dark at ZT7, while for FRL control plates were kept under WL for the 15-min duration of the FRL pulse. For all samples, the temperature was kept constant at 19°C for the duration of the light pulse. Samples were then imaged immediately for up to 1 h following the cessation of the light pulse.

### Confocal microscopy

The Leica Zeiss 710 confocal laser scanning microscope with Plan-Apochromat 63×/1.4 Oil DIC M27 objective and Zen 2011 SP4 confocal software (Leica, Wetzlar, Germany) was used to collect images. Arabidopsis seedlings were submerged in deionized water on clear white slides. For all imaging reported here, the YFP fluorochrome was excited at 514 nm and emission detected between 525 and 615 nm. The pinhole was set to airy one for all constructs. The same laser setting was used for all images collected during this work, regardless of the mutant background: laser power = 4%, master gain = 695, digital gain = 2.6, and digital offset = 23.40. All images were collected as Z-stacks, with a Z-stack slice depth of 0.4 µm. All presented images are min/max projections of compiled Z-stacks.

Foci were counted from compiled Z-stacks projected as a 2.5D min–max image in the Zen 2011 SP4 software. These counts were then validated by scoring each image of the compiled Z-stack for foci. To calculate the N/C ratio, compiled Z-stacks were imported into ImageJ (version 1.51W) (Schneider et al., 2012) where the total image signal and nuclei signal were measured for each image through the use of hand-drawn perimeters. Background values were also calculated, and the mean of these values were subtracted from the total image and individual nuclei signal. The mean nuclei signal was then calculated for all images of that respective treatment, while the total image signal for each image was normalized by the mean number of nuclei per image for that respective condition. The N/C ratio was then calculated from the mean nuclear and normalized total signal values. Images were collected on at least two separate occasions, with similar biological responses observed each time. The sample size is described in the figure legend, all statistical analysis was performed in R studio (version 1.4.1717, rstudio.com) using the R version 3.6.1.

### RT-qPCR

Ws-2 *LHY::LUC* (WT), *35S::YFP:ELF3* (*elf3-4*), and *35S::YFP:ELF3* (*elf3-4/phyB-10*) seedlings were grown as described in the light pulse section described above. On the seventh day, seedlings were either transferred to the dark or pulsed with 25 µmol of RL for 3 h at ZT7 before ∼100 mg of seedlings were harvested and snap-frozen in liquid nitrogen at ZT10. RNA was harvested using the Qiagen RNeasy Plant Mini Kit before DNase treatment and subsequent clean-up was performed using the Zymo clean and concentrator kit. Then, 1 µg of cDNA was generated using SuperScript IV (ThermoFisher Invitrogen, Waltham, MA, USA) and diluted to a concentration of 1.25 ng/µL. Manufactures’ recommendations were followed for all protocols.

All reverse transcription-quantitative PCR (RT-qPCR) was performed on an ABI StepOnePlus machine using the StepOne Plus version 2.3 software package. Fast SYBR GREEN (Thermo Fisher Applied Bioscience, Waltham, MA, USA) was used for all qPCRs. The efficiencies of primers were determined for an annealing temperature of 60°C. Primers sequences are described in Supplemental material (Supplemental Table S2). The presented data is the mean of at least two technical replicas with error bars representing standard deviation among the technical repeats. The presented results are representative of two independent biological replicas.

### Western blotting

Seeds of *35S::GFP:ELF3* (Col-0) were surface sterilized, plated onto 1× MS plates with 0.8% w/v agar, 0.25% w/v sucrose, and 0.5 g/L MES with a pH of 5.7. Seeds were subsequently stratified for 3 days before being transferred to a short-day growth cabinet with 125 µmol of WL and a constant temperature of 22°C. Seedlings were subsequently grown for 17 days before light treatments (as described above) were started at ZT7 on Day 18. The seedlings were thoroughly grinded and total proteins were extracted with the buffer containing 50 mM Tris–HCl, pH 7.5, 150 mM NaCl, 1 mM EDTA, 0.1% Nonidet P-40 (v/v), 1 mM PMSF, 1 mM DTT, 2 mg/mL Chymostatin, 2 mg/mL Leupeptin, 2 mg/mL Pepstatin, 2 mg/mL Aprotinin, 50 mM MG132, 50 mM MG115, 50 mM ALLN, 2 mM NaF, and 2 mM Na3VO4. About 10 µL GFP tagged ELF3 proteins (added with SDS loading buffer) were separated by 8% SDS-PAGE. Immunoblotting was performed using a 1:2,000 dilution of the primary anti-GFP antibody (Abcam, Cambridge, UK; ab6556) or a 1:2,000 dilution of the primary anti-Actin (EASYBIO, Seoul, Korea). The 1:3,000 horseradish peroxidase (HRP)-linked anti-rabbit IgG was used as secondary antibodies. Experiments were repeated twice, with similar results observed on each occasion.

## Accession numbers

Accession numbers are as described by TAIR (https://www.arabidopsis.org/) as follows: EARLY FLOWERING3 (ELF3): AT2G25930; phyB: AT2G18790; LUX ARRYTHMO (LUX): AT3G46640; EARLY FLOWERING4 (ELF4): AT2G40080; GI: AT1G22770; TIMING OF CAB EXPRESSION1 (TOC1): AT5G61380; PRR9: AT2G46790; PROTEIN PHOSPHATASE2A (PP2A): AT1G13320.

## Supplemental data

The following materials are available in the online version of this article.

**Supplemental Figure S1.** The cellular localization of ELF3 is light responsive.

**Supplemental Figure S2.** Light treatments do not influence the stability of ELF3.

**Supplemental Figure S3.** Equal ratios of RL and BL induce a wide range of responses.

**Supplemental Figure S4.** Monochromatic BL pulses induce foci formation independently of light intensity.

**Supplemental Figure S5.** The nuclear accumulation of ELF3 is not changed by 10 or 15 µmol RL pulses.

**Supplemental Figure S6.** Photoactivated phys are required for ELF3 nuclear and sub-nuclear accumulation.

**Supplemental Table S1.** Genotyping primers.

**Supplemental Table S2.** qPCR primers.

## Supplementary Material

kiac072_Supplementary_Data

## Notes

J.R. and S.J.D. conceived the original research plans. L.W. and S.J.D. supervised the experiments. J.R. and C.S. performed the experiments. J.R. wrote the article with contributions of all the authors. S.J.D. agrees to serve as the author responsible for contact and ensures communication.

The author responsible for distribution of materials integral to the findings presented in this article in accordance with the policy described in the Instructions for Authors (https://academic.oup.com/plphys/pages/general-instructions) is: Seth J. Davis (seth.davis@york.ac.uk).
