# Supplementary material for: Cellular localization of Arabidopsis EARLY FLOWERING3 is responsive to light quality
Source: Plant Physiol. 2022 Feb 22;190(2):1024–36. doi: 10.1093/plphys/kiac072 (PMC9516731; doi:10.1093/plphys/kiac072)
Supplement: kiac072_Supplementary_Data [file kiac072_supplementary_data.pdf]

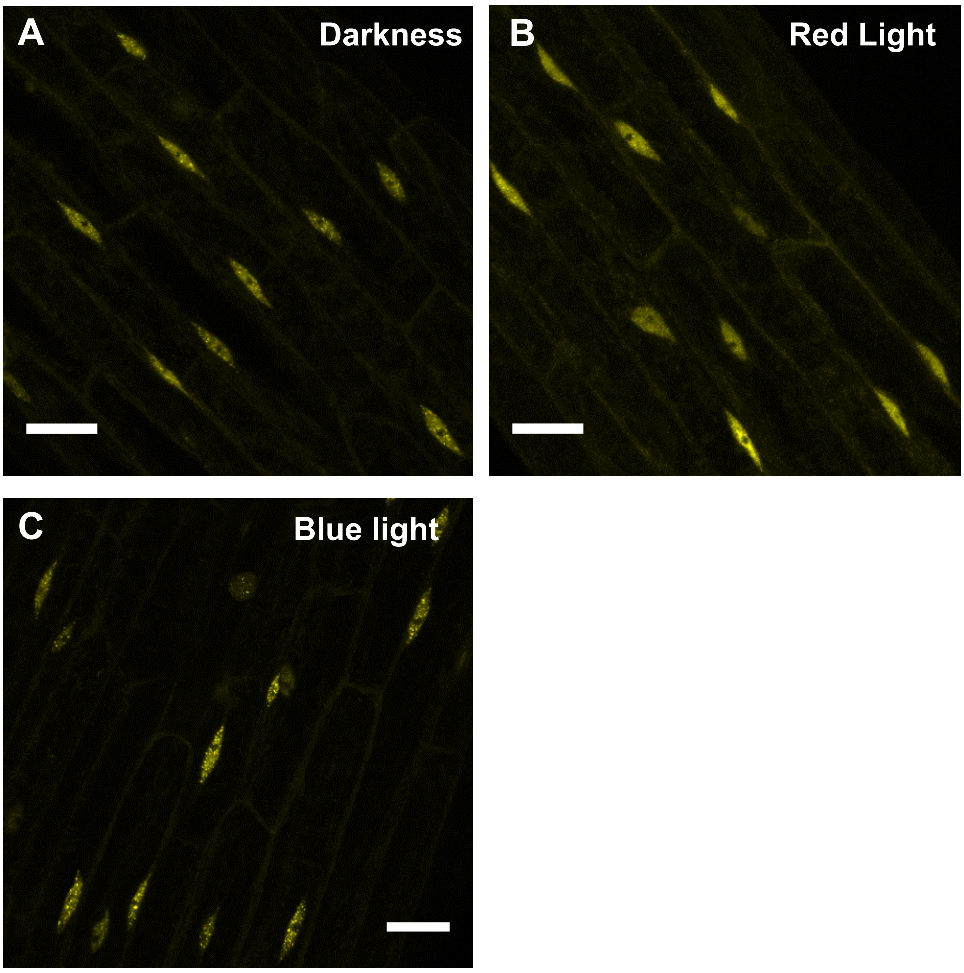

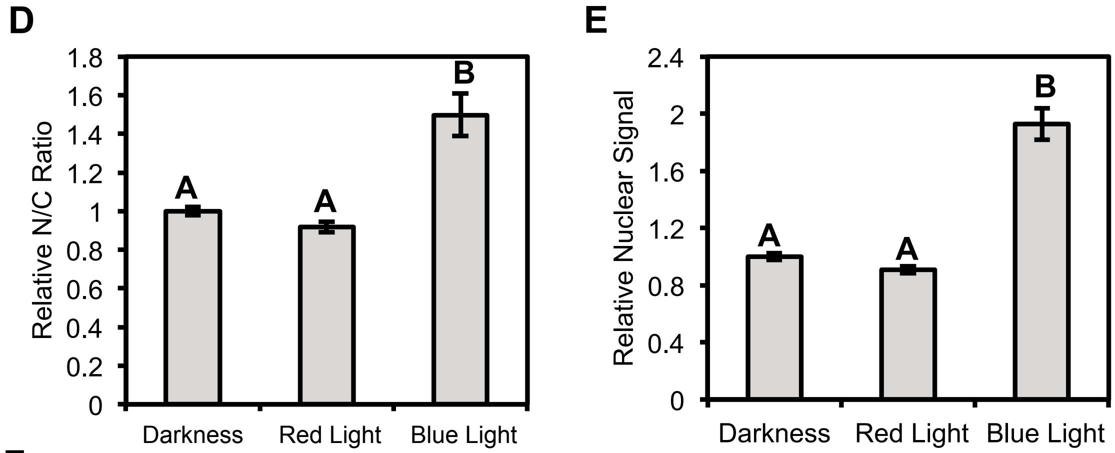

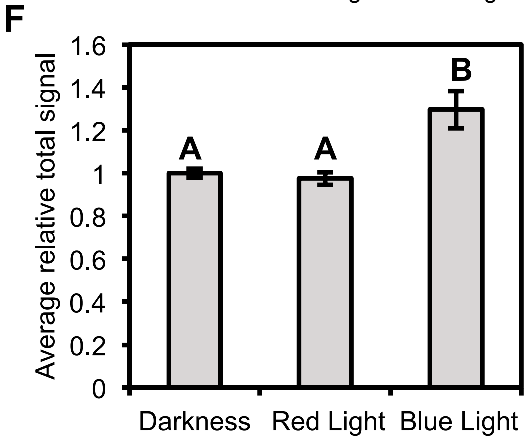

Supplementary Figure 1 - The cellular localization of ELF3 is light responsive. The cellular localization of *35S::YFP:ELF3* in hypocotyl cells after samples were either transferred to the **(A)** dark or after a 25 μmol **(B)** red or **(C)** blue-light pulse. All light pulses were started at ZT7 (short-day 6/18 photoperiods) and applied for three-hours before imaging was started at ZT10 for up to one hour. As a control, samples were transferred to the dark at ZT7. Scale bars equal 25 μm. **(D)** Relative nuclear/cytoplasmic (N/C) ratio, **(E)** relative nuclear signal and **(F)** relative total signal of ELF3 under the respective light regimes. All data was made relative to the values for ELF3 in the dark. A minimum of four images were analyzed for each light treatment. Error bars are standard error of the mean. A one-way ANOVA with a tukey-HSD posthoc test was used to determine significant difference, different letters signify a significant difference of $p < 0.001$.

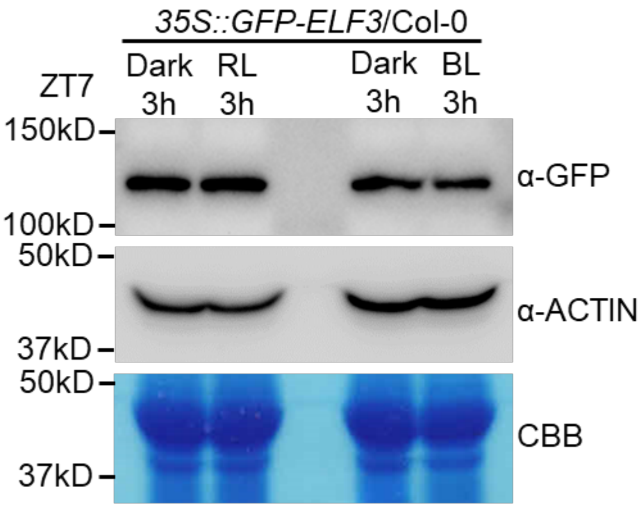

Supplementary Figure 2 – The stability of ELF3 is unchanged by 3-hour pulses of blue or red light. The stability of ELF3 in *35S::GFP:ELF3* (Col-0) was analyzed in 18-day old plants. Plants were grown under short-day photoperiods for 17-days before plants were pulsed with 25 µmol of red (RL) or blue light (BL) for three-hours. As a control, plants were moved to the dark. The intensity and timing of the light pulses were as described for the cellular localization experiments. CBB, Coomassie brilliant blue dye.

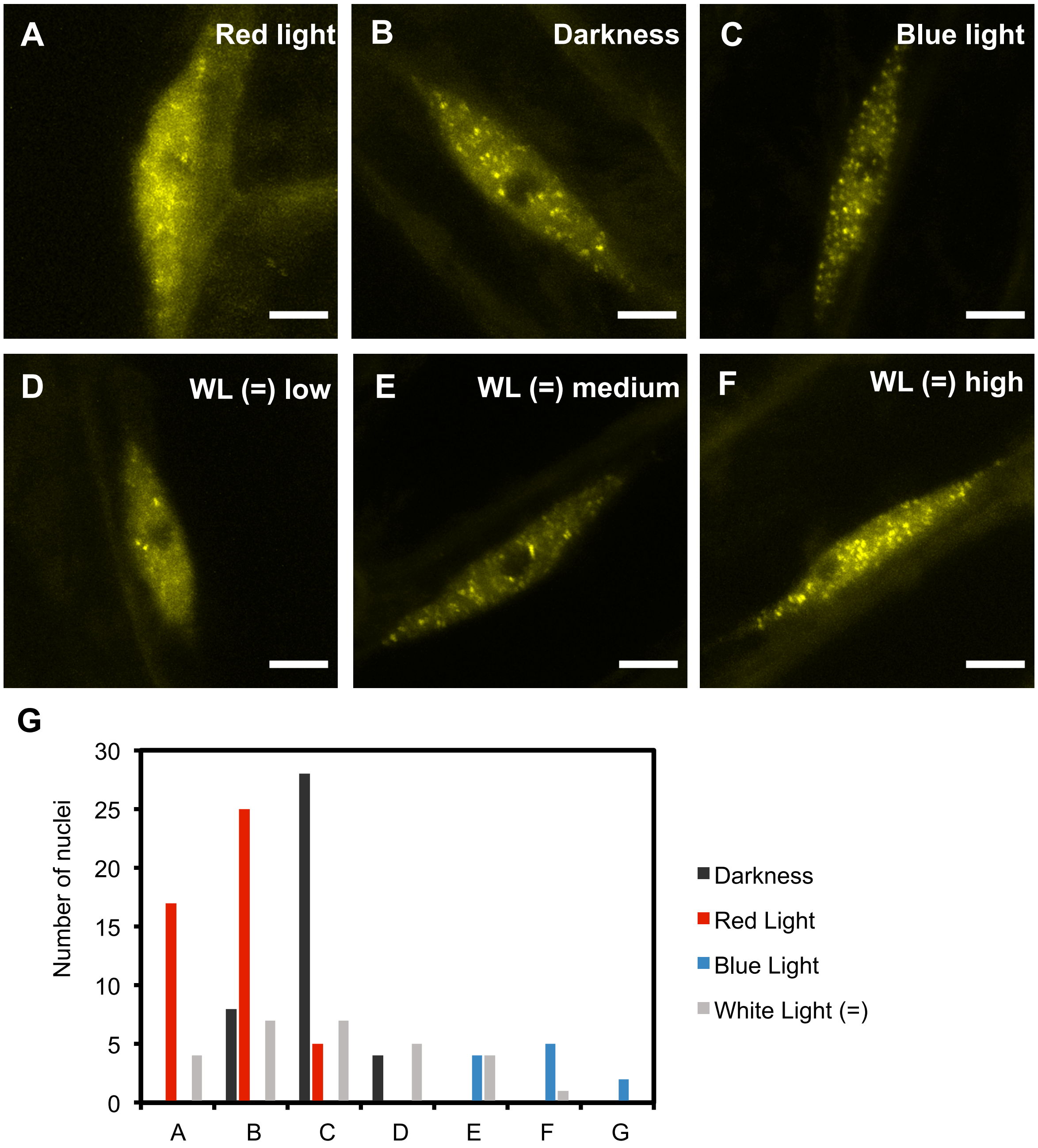

Supplementary Figure 3 - Equal ratios of red and blue light induce a wide range of foci responses. Nuclei of seven-day old seedlings expressing *35S::YFP:ELF3* (*elf3-4*) were imaged at ZT10 after various light pulses that were started at ZT7. Nuclei were pulsed with **(A)** 25 μmol of red light (RL), **(B)** transferred to the dark, **(C)** pulsed with 25 μmol of blue light (BL) or **(D-F)** pulsed with white light (WL: 25 μmol of RL and 25 μmol of BL). (**D-F**) The spectrum of foci responses seen under an equal RL:BL intensity white light pulse; **(D)** similar foci response to monochromatic RL, **(E)** similar to response to samples in the dark and **(F)** similar response to samples pulsed with monochromatic BL. **(G)** Histogram plot of the number of nuclei with a certain number of foci. Bins on the X axis represent: A = 0 – 4, B = 5 – 9, C = 10 – 14, D = 15 – 19, E = 20 – 24, F = 24 – 29, G 30 – 35 foci per nucleus. Data for darkness, monochromatic RL and BL is the same as presented earlier (figure 1), while the white light data is the same as presented in figure 2.

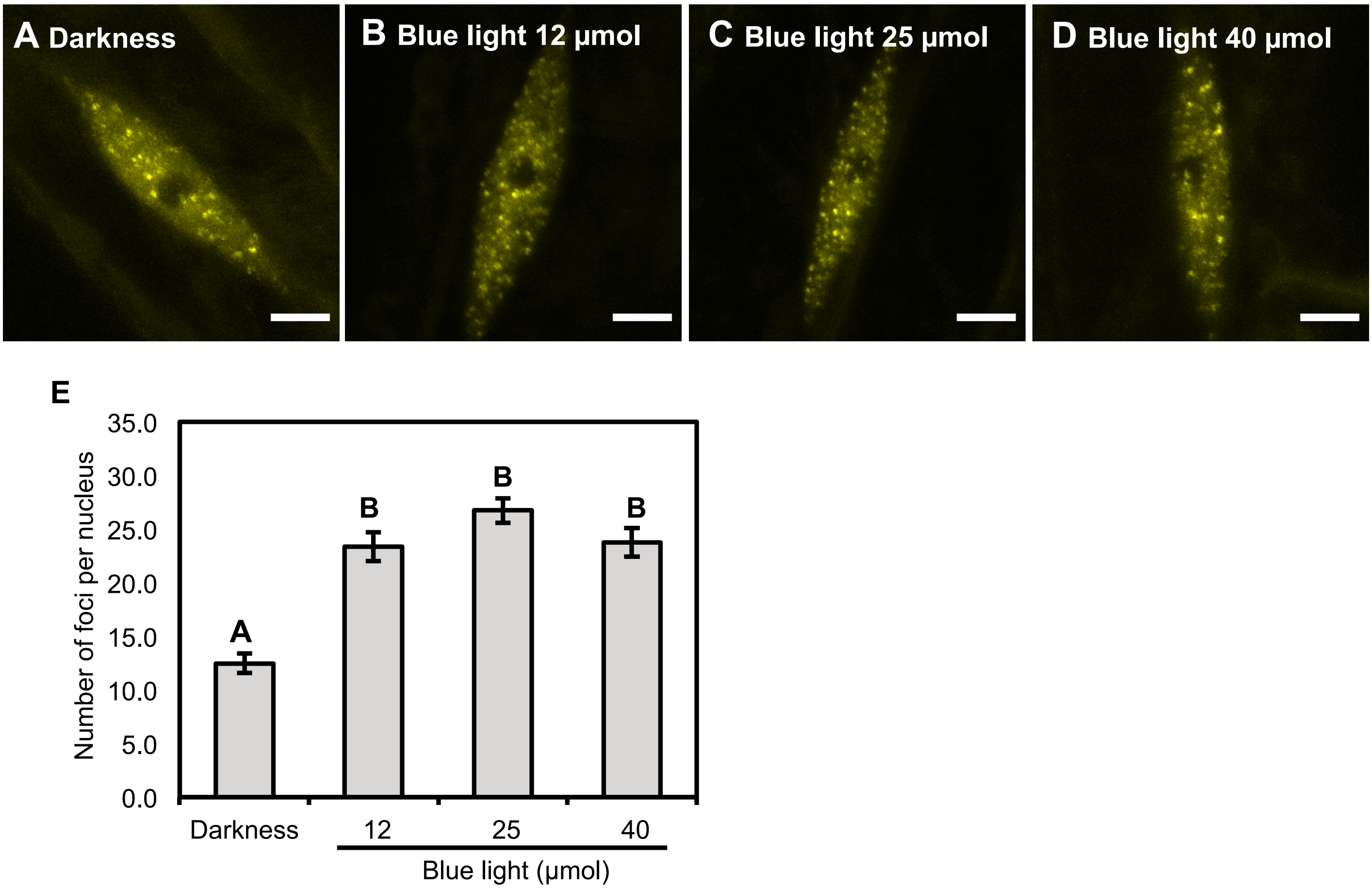

Supplementary Figure 4 - Monochromatic blue light promotes foci formation independently of light intensity. Nuclei of seven-day-old seedlings expressing *35S::YFP:ELF3* (*elf3-4)* were imaged at ZT10 after being either transferred to the (**A**) dark or pulsed with **(B)** 12 μmol, **(C)** 25 μmol or **(D)** 40 μmol of blue light (BL) for three hours starting at ZT7 (1-hour before dawn, short day photoperiods). **(E)** Mean number of foci per nucleus. All imaging was repeated twice, with the presented data a combination of these independent repeats. Similar results were obtained each time. In total, a minimum of eight nuclei were analyzed for each light treatment. Error bars are standard error of the mean. Significance was determined by a one-way ANOVA with a tukey HSD posthoc test. Different letters signify a significant difference of $p < 0.001$. Scale bars in all images are 5 μm. The darkness and BL25 data are the same as first presented in figure one of the main text.

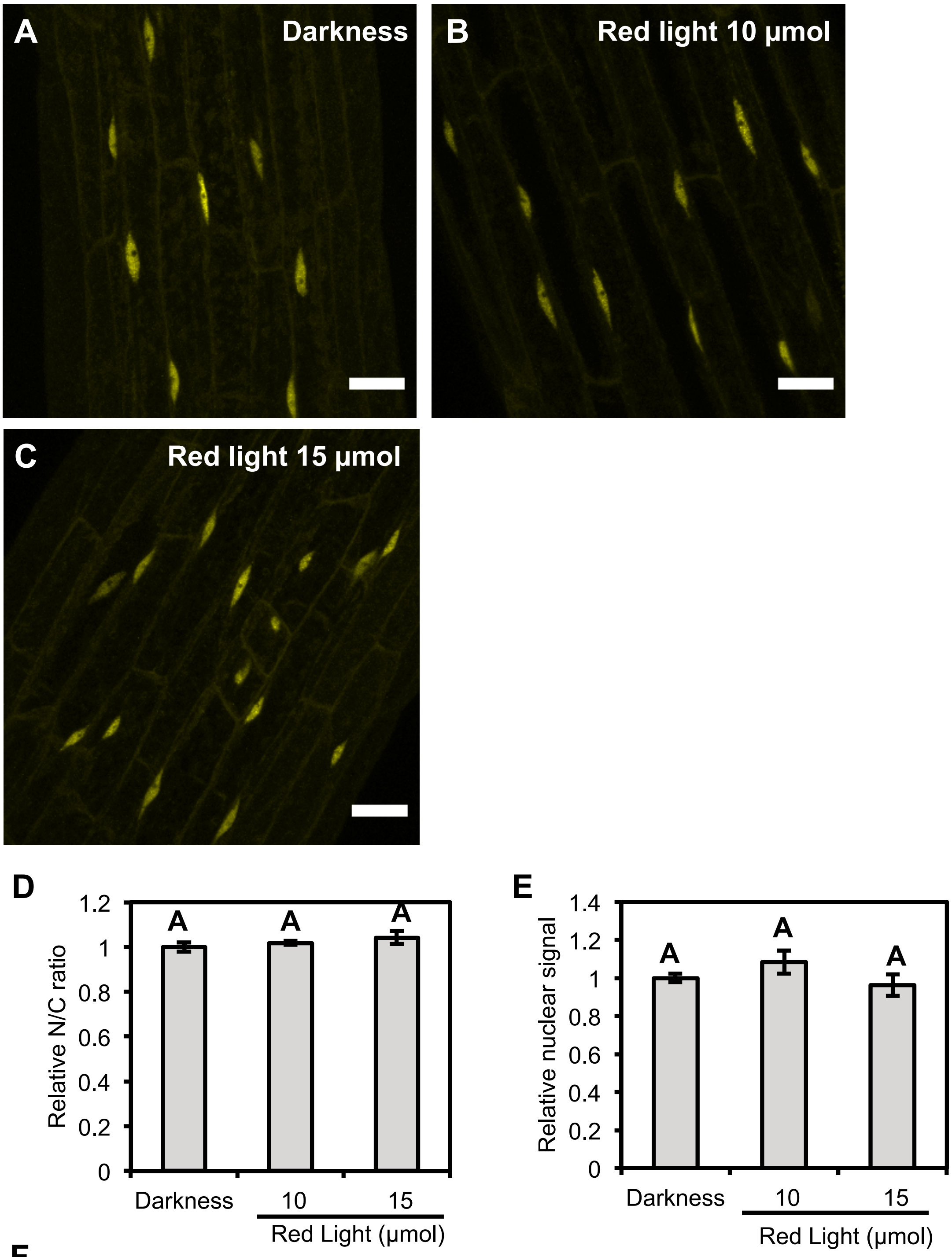

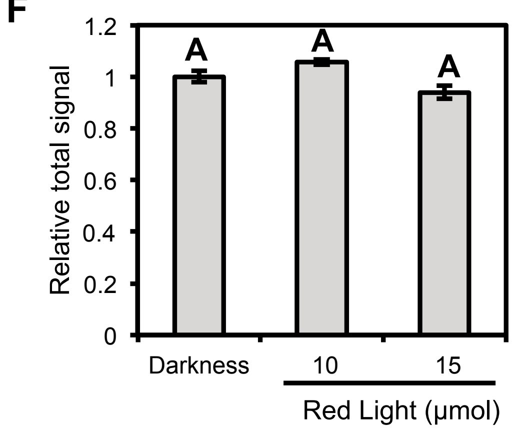

Supplementary Figure 5 - The nuclear accumulation of ELF3 is not affected by 10 or 15 µmol red light pulses. The nuclear localization of *35S::YFP:ELF3* (*elf3-4*) in the **(A)** dark, or after a **(B)** 10 *µ*mol, or **(C)** 15 *µ*mol red-light (RL) pulse. Scale bars equal 25 *µ*m. **(F)** The relative nuclear/cytoplasmic (N/C) ratio, **(G)** relative nuclear signal and **(H)** relative total signal of *35S::YFP:ELF3* (*elf3-4*) under the respective light treatments. All data were made relative to the values of ELF3 in the dark. All light pulses were started at ZT7 (8/16 short-days) and carried out for three-hours before seedlings were imaged at ZT10. A minimum of four images were analyzed in total for each light treatment. Error bars are standard error of the mean. Significance was determined by a one-way ANOVA with a tukey HSD posthoc test. Same letters indicate no significant difference. The darkness dataset is the same as first presented in Supplemental Figure S1.

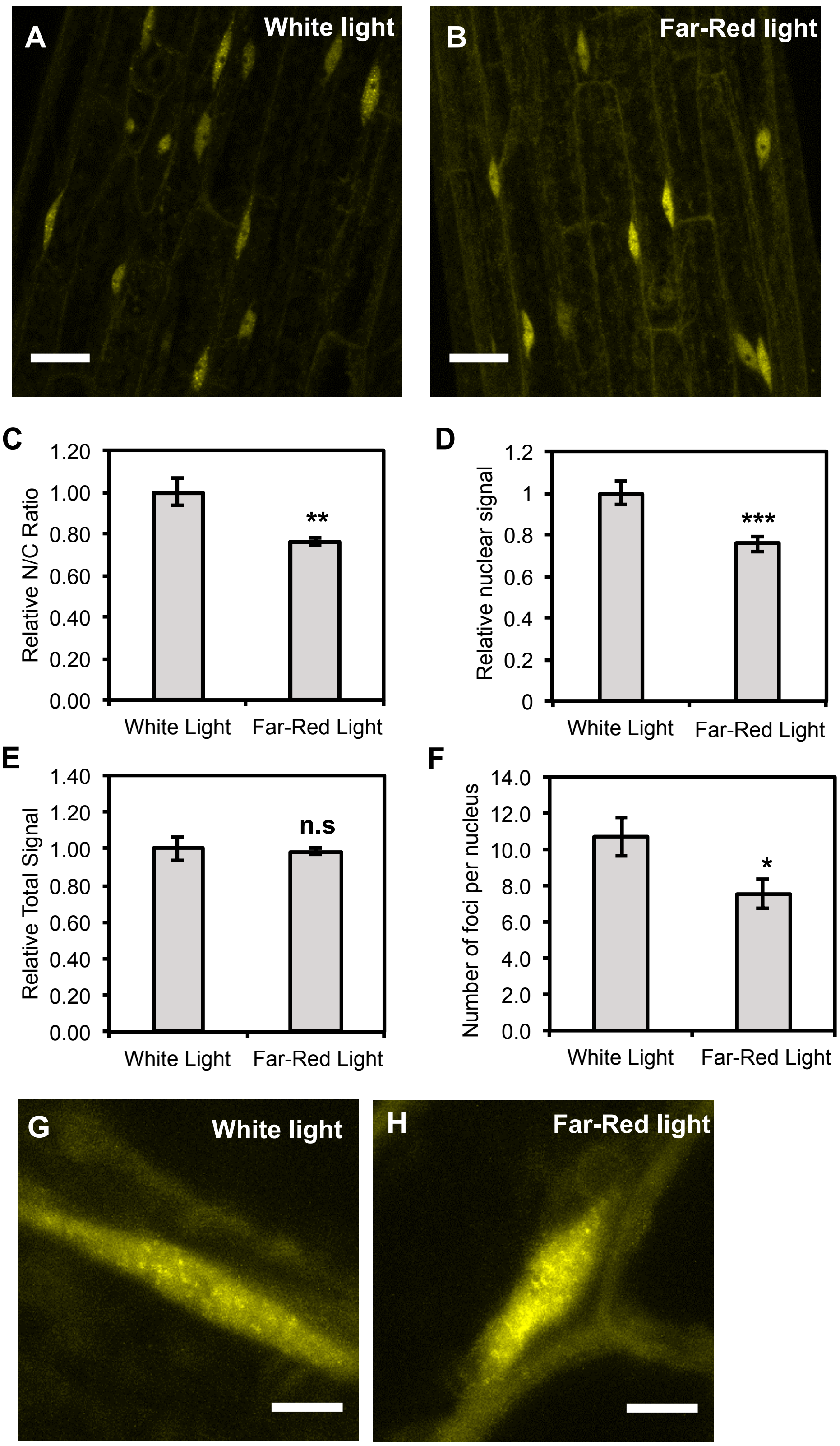

Supplementary Figure 6 - Photoactivated phys are required for the nuclear accumulation of ELF3. The localization of *35S::YFP:ELF3* at ZT7 after either exposure to (**A, G**) 85 μmol of white light (WL) or (**B, H**) a 110 μmol far-red light (FRL)-pulse. The FRL-pulse was applied for 15-minute starting at ZT7 (one-hour before dawn, 8/16 photoperiods). Scale bars in **(A, B)** are 25 μm, while scale bars in **(G, H)** are 5 μm. **(C)** The relative nuclear/cytoplasmic (N/C) ratio, **(D)** relative nuclear signal, **(E)** relative total signal and **(F)** number of foci per nucleus under the respective light treatments. Error bars are standard error of the mean. A minimum of (C-E) three or (F) 10 images were analyzed for each light treatment. Statistical significance was determined by a two-way, unpaired T test . n.s = no significant difference, * = $p < 0.05$, ** = $p < 0.01$ and *** = $p < 0.001$.

Supplementary table 1 - Genotyping primers

Genotype	Sequence		Amplicon Size
elf3-4	F	TGCAGATAAAGGAGGGCCTA	Wild type: 146 bp<br>Mutant: 140 bp
	R	ATGGTCCAGGATGAACCAAA	
phyB-10 (Exon 1)	F	CTATGGGGAAGTCTCTGGTT	Wild type: 2 kb<br>Mutant: No band
	R	CTAATATGGCATCATCAGCATC	
phyB-10 (Exon 4)	F	AATGGCGTGTCCAGGTGAAG	256 bp
	R	CTAATATGGCATCATCAGCATC	
35S::YFP:ELF3	F	GGGCATCGACTTCAAGGAGGAC	1307 bp
	R	ACAAAGCCACCTGACCTTGCA	

Supplementary table 2 - qPCR primers

Gene Target	Sequence		Source
PP2A	F	TATCGGATGACGATTCTTCGTGCAG	Herrero *et al.,* 2012[1].
	R	GCTTGGTCGACTATCGGAATGAGAG	
PRR9	F	GGAAGTGGTGCTCAGGCTAT	This study.
	R	GCTCAGGACCAACACACTC	
LUX	F	GCTTCGGATAAGCTCTTCTCTTC	Kolmos *et al.,* 2009[2].
	R	ATAAACTGGCATCTGCATCATCT	
TOC1	F	GTTGATGGATCGGGTTTCTC	Lee *et al.,* 2019[3].
	R	TCATGACCCCATGCATATAG	
GI	F	AGCAGTGGTCGACGGTTTATC	Nieto *et al.,* 2015[4]
	R	ATGGGTATGGAGCTTTGGTTC	

1 DOI: 10.1105/tpc.111.093807
2 DOI: 10.2976/1.3218766
3 DOI: 10.1038/s42003-019-0377-7
4 DOI: 10.1016/j.cub.2014.10.070
